# Evaluation of the Carbon Footprint, Water Footprint, Nutrient Profiles and Cost of Sustainable Menus Planned With Digital Modeling

**DOI:** 10.1002/fsn3.70977

**Published:** 2025-09-18

**Authors:** Nida Nur Adiyan, Yasemin Beyhan, Taygun Dayi

**Affiliations:** ^1^ Department of Nutrition and Dietetics, Faculty of Health Sciences Hasan Kalyoncu University Gaziantep Türkiye; ^2^ Department of Nutrition and Dietetics, Faculty of Health Sciences Near East University Nicosia Cyprus

**Keywords:** cost, digital modeling, ecological impact, nutrient profiling, sustainable menus

## Abstract

This study aimed to evaluate the sustainability of digital program menus (DPMs) and compare them with the Turkish National Menu Planning Guide (TNMPG) and internet‐accessible menus (IAM). A menu planning program was developed in Microsoft Excel, which also calculates the sustainability criteria (carbon footprint, water footprint, NRF 9.3, SAIN‐LIM and cost) of the menus with the recommendations of the TNMPG. The digital menus were compared with the sustainability criteria of the TNMPG sample menus and menus accessed from the internet. DPM was found to be similar to the sample menus of the TNMPG in terms of sustainability features. All the meals on the menus were similar in terms of their carbon footprint (*p* > 0.05), whereas the II‐type meals of the kindergarten menu of DPM and the IV‐type meals of the hospital menu of IAM were found to have greater water footprints (*p* < 0.05). Some meals (II/III) in DPM had higher NRF‐9.3 scores (*p* < 0.05). The I‐type meals of the kindergarten and prison menus of DPM and the II‐type meals of the prison menu were found to have significantly higher costs (*p* < 0.05). Digitalization could provide great benefits in service, as digital menus in food services can be prepared in a very short time, and sustainability criteria can be easily achieved.

## Graphical abstract

A menu planning program was developed in Microsoft Excel, which also calculates the sustainability criteria (carbon footprint, water footprint, NRF 9.3, SAIN‐LIM and cost) of the menus with the recommendations of the TNMPG. The digital menus were compared with the sustainability criteria of the TNMPG sample menus and menus accessed from the internet. In our study, it was shown that it is possible to plan sustainable menus in accordance with nutritional principles for FS and to evaluate the sustainability of these planned menus by calculating the ecological impact factors, nutritional pattern profiles and costs in a short time. When the DPM was compared with the NMPG menus, the DPM and NMPG menus were similar in terms of sustainability qualities.

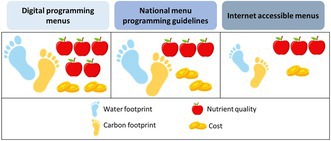

## 1 Introduction

Nutrition, at the core of Maslow's hierarchy of needs, is necessary for all people to survive (Hale et al. 2019). Adequate and balanced nutrition forms the basis of human health and happiness, physical and cognitive development, and economic production. This situation has a very important role in breaking the cycle of poverty between generations. A report published by the UNFPA (United Nations Population Fund) predicts that the world population will reach 9.7 billion in 2050 and 10.4 billion in 2100 (UNDP 2020). Global food production needs to increase by 1%–2% per year to provide sufficient food to the ever‐increasing population (UNFPA 2022). With the increase in food production, greenhouse gas emissions increase, creating a climate change problem. Climate change is strongly linked to the food chain and food security (TUBER 2022). Therefore, sustainable nutrition has gained importance in many aspects, such as ensuring food security and preventing climate change.

The concept of sustainable nutrition was defined by the Food and Agriculture Organization in 2010 as a diet that has low environmental impact; is accessible, affordable, safe, fair, and culturally acceptable; and supports the health and well‐being of individuals in all aspects (FAO 2010). Sustainable diets are not just a form of nutrition but also a lifestyle that encompasses all components of a healthy and sustainable life with dimensions such as health, biodiversity, equality, cultural heritage, food security, and locality (TUBER 2022). Sustainable nutrition emphasizes that the food chain should be sustainable at all stages, that it should provide food and nutrient requirements, and that food production systems should be in solidarity (Guimarães et al. 2024; Maynard et al. 2020).

In institutions and organizations where food services (FS) are provided, which are places where food prepared by others outside the home is consumed, the service covers all processes from the purchase of food to its presentation to the consumer. To ensure sustainable nutrition in these institutions and organizations, each stage that constitutes food systems must be suitable in terms of sustainability (Blondin et al. 2020; Derqui et al. 2020; Perez‐Neira et al. 2021).

The success of FS depends on the planned and implemented menus. Menu planning in these businesses is a complex process affected by issues such as ensuring healthy nutrition, season, budget, consumer satisfaction, color, consistency, and taste harmony of the dishes served together (Lima et al. 2023). In addition, to ensure sustainability, the menu planning process becomes even more complex with the addition of factors such as the selection of foods with the least environmental impact, providing variety, being budget‐friendly, and having high nutritional value (Baygut and Bilici 2021; Carletto et al. 2023; dos Santos et al. 2022). Dieticians who manage this complex structure need to be able to access quantitative data (carbon footprint, water footprint, nutrient profile, etc.) important for sustainability clearly and quickly because of the limited time they have while designing sustainable menus. Therefore, the inclusion of digital applications that offer convenience in every field of FSs today, in addition to the convenience of designing sustainable menus, has become important because it provides convenience, such as saving time and tracing in data (Aytekin‐Sahin et al. 2024).

With this in mind, in the presented study, the data (carbon footprint, water footprint, nutritional profile and cost) that are important for the sustainability of the menus created with the menu planning program developed for different target groups (preschool‐age children, hospitals, nursing homes, prisons, workplaces‐workers) and the menus consisting of three types of meals for the one‐month autumn period obtained from two different sources (National Menu Planning Guide and accessed from the internet) were evaluated.

## 2 Materials and Methods

### 2.1 Time, Ethical Approval, and Design of the Study

This study was conducted between December 2022 and October 2023. To conduct the study, permission was obtained from the Hasan Kalyoncu University Non‐Interventional Scientific Research Ethics Committee, dated 12.12.2022 and numbered 2022/139. In the presented study, to evaluate the menus planned with the digital menu planning program and compare their sustainability, sample menus in the “National Menu Planning and Implementation Guide” (Türkiye Ministry Health 2020) for the fall semester consisting of three types of meals a one‐month for these five target groups (preschool children, hospitals, nursing homes, prisons, workplaces‐workers) and menus offered by catering companies accessed via the internet were included (5 target groups × 3 sources: 15 menus in total). The menus, whose target group is patients, consists of four types of meals, and in addition to the menus planned through the program, the fourth type of meal was manually selected and placed.

Design of the digital menu planning program in our study, a digital menu planning program was developed using MS Excel to plan 1‐month autumn season menus consisting of three different meals for target groups (in Figures [Link], [Link], [Link], [Link], [Link], [Link]). The target groups included preschool children, hospitals, nursing homes, prisons, and workplaces‐workers. The database created in the development of the program includes all foods included in the menu, nutrient and energy contents of these foods, the carbon footprint and water footprint factors, the needs of target groups and recipes, nutritional profile algorithms (NRF 9.3 and SAIN‐LIM), and 2023–2024 Türkiye meal costs (g/product cost). Then, a decision mechanism was created to plan menus suitable for target groups, and the data were processed into the program and used appropriately. The developed prototype program was presented to a panel of 5 expert dietitians, and the program was revised and finalized in line with their opinions and suggestions. The total energy (kcal), carbohydrate (g), protein (g), and fat (g) amounts of these planned menus and the carbon footprint, water footprint, NRF 9.3, and SAIN‐LIM values of each meal type in the menu were obtained as outputs.

In addition, the menus planned in this program consist of three types of meals, and if a fourth dish is added to the program or any inconsistency is noticed, the opportunity to select/applicate manual interventions is provided. In this way, the program also allows manual changes to be made to the meals on the menu.

### 2.2 Carbon Footprint and Water Footprint Calculations for Menus

Since there are currently no data on greenhouse gas emissions and the carbon footprint factors of foods produced in Türkiye, calculations were performed in this study via the “standard average carbon footprint factors” compiled because of meta‐analyses of various studies worldwide (Clune et al. 2017; Heller and Keoleian 2015; Tilman and Clark 2014). Carbon footprint factors for each food are determined in the literature in kg/product, and according to the recipes of the meals included in the menus evaluated in our study, the carbon footprint factors of the foods were converted to g/product to calculate the carbon footprint of one portion of the meals.

To calculate the water footprint of each of the foods, agricultural products and animal products included in the menus in our study, the water footprint factors (m^3^/ton) obtained from the literature studies based on the “Water Footprint Standard” developed by the WFN were used (Mekonnen and Hoekstra 2011, 2012).

Since the spices and various flavorings in the recipes are included in trace amounts in the meals and since some specific products do not have carbon and water footprint factors, these were not included in the calculation.

### 2.3 Evaluation of Nutrient Profiles of Menus

In our study, the nutrient profiles of the menus were evaluated with the internationally used and validated SAIN‐LIM model and the “Nutritional Rich Food” (NRF9.3) model (Pahlow et al. 2015; Drewnowski 2005, 2009, 2010). The calculation algorithm of the NRF 9.3 index is available in the literature (Drewnowski 2009, 2010), and the SAIN‐LIM score was defined and calculated in MS Excel (Darmon et al. 2009). In this study, when the SAIN and LIM scores are evaluated,

If the SAIN score is ≥ 5 and the LIM score is ≤ 7.5, “recommended foods”: class 1;

If SAIN score is < 5 and the LIM score is < 7.5, “neutral foods”: class 2;

If SAIN score is ≥ 5 and the LIM score is ≥ 7.5, “foods that should be consumed less”: class 3;

If the SAIN score is < 5 and the LIM score is ≥ 7.5, “foods that should be limited”: class 4.

A high class score indicates low nutritional value, whereas a low class score indicates high nutritional value.

### 2.4 Calculation of Total Cost Values of Menus

For the cost calculation of the foods included in the five‐day menu, the websites of institutions/organizations were used (Türkiye Trade Minitry 2022; Türkiye Soil Products Office 2022; Meat and Milk Institution 2022; Adana Commodity Exchange 2023). The one‐portion values of each menu were determined in accordance with the “Standard Food Recipes” (Merdol 2018) and “TNMPG” (Türkiye Ministry Health 2020), and the total cost values were calculated. Other cost factors were not included in the cost calculation; only the meals' raw material costs (g/product cost) were determined. The cost was calculated in dollars at the exchange rate at the time the study was conducted.

### 2.5 Statistical Analysis

The data were analyzed and evaluated with the SPSS Statistics 27.0 program. The means and standard deviations were used for descriptive statistics. Data distributions were examined with the Kolmogorov–Smirnov test and the Shapiro–Wilk normality test. Kruskal–Wallis variance analysis and the Mann–Whitney *U* test were used to compare energy and nutritional values and sustainability criteria between menus. The relationships between nutrient profiles (NRF 9.3 and SAIN‐LIM) were tested via the Spearman correlation coefficient. The results were at the 95% confidence interval, and *p* < 0.05 was taken as the basis for the significance level.

## 3 Results

The nutrient, energy, and macronutrient contents (g/per menu) of a meal from menus obtained from three different sources are shown in Table 1. No significant difference was found between the menus in terms of average energy, protein, or carbohydrate content (*p* > 0.05), but the difference was significant in terms of fat content (*p* = 0.029).

**TABLE 1:** Average food, energy, and macronutrient compositions of menus.

	DPM	NMPG menus	IAM	*p* *
Food type (g/per menu)				
Red meat	76.8	65.0	51.2	
Poultry	19.4	31.9	54.8	
Fish	14.05	17.4	0.2	
Milk or milk products	80.7	114.8	113.3	
Eggs	9.4	5.8	7.7	
Legumes and pulses	14.7	39.4	35.2	
Cereals	43.5	46.5	56.5	
Vegetables and fruits	423.8	346.9	229.1	
Oils	17.7	17.6	20.3	
Nuts	0.7	1.9	1.6	
Energy and macronutrients				
kcal/per menu	720.0	803.3	776.2	0.192
g protein/per menu	46.3	48.3	48.9	0.851
g fat/per menu	**29.1** ^**a**^	**32.7** ^**b**^	**32.5** ab	**0.029**
g carbohydrate/per menu	61.7	71.1	65.9	0.168

*Note:* Parameters with different exponent letters (a, b) are significantly different from each other. *p* < 0.05.

Abbreviations: DPM, Digital Programme Menus; IAM, internet‐accessible menus; NMPG, National Menu Planning Guidelines.

* Kruskal‐Wallis Test.

^ab^ Mann‐Witney *U* Test.

When the ecological effects of the menus were examined by dividing them into target and meal groups (Table 2), the highest carbon footprint and water footprint values were in the first type meals, which are the main courses. Although the average (x̄ ±SD) carbon footprint of DPM was found to be greater than that of the other menus, no statistical difference was found in terms of the carbon footprint between the menu sources (*p* > 0.05). The I‐ and II‐type meals in the three menu sources were similar in terms of their water footprint (*p* > 0.05). However, the water footprint of the II‐type meals of the DPM, whose target group was preschool‐age, was found to be greater than that of the other menu sources (*p* = 0.002). In addition, the water footprints of the IV‐type meals in the DPM and the NMPG hospital menus were similar (*p* > 0.05) and lower than those of the menus with internet access (*p* = 0.039).

**TABLE 2:** The mean carbon and total water footprints of menus.

Target group	Meal types	DPM	NMPG menus	IAM	*p* *	DPM	NMPG menus	IAM	*p* *
		Mean carbon footprint (kg/CO_2_ equivalent) ± SD				Mean total water footprint (m^3/^ton) ± SD			
Preschool‐age	I‐course	0.0444 ± 0.0728	0.0242 ± 0.0158	0.0207 ± 0.0082	0.116	1.4019 ± 0.6180	1.1677 ± 0.7326	1.1788 ± 0.3356	0.438
Hospitals***		0.0256 ± 0.0129	0.0285 ± 0.0167	0.0263 ± 0.0138	0.858	1.3016 ± 0.5890	1.4093 ± 0.8127	1.3868 ± 0.5150	0.896
Workplace‐worker		0.0327 ± 0.0108	0.0272 ± 0.0155	0.0272 ± 0.0147	0.168	1.5610 ± 0.4350	1.3825 ± 0.7259	1.5195 ± 0.6576	0.563
Nursing home		0.0256 ± 0.0118	0.0245 ± 0.0154	0.0221 ± 0.0158	0.336	1.2878 ± 0.5414	1.2398 ± 0.7060	1.2220 ± 0.6806	0.910
Prison		0.2850 ± 0.0164	0.0223 ± 0.0103	0.0187 ± 0.0106	0.115	1.4017 ± 0.7626	1.2128 ± 0.4636	1.0786 ± 0.4752	0.210
Preschool‐age	II‐course	0.0055 ± 0.0047	0.0028 ± 0.0024	0.0032 ± 0.0022	0.084	**0.3315 ± 0.2182** ^**a**^	**0.1939 ± 0.0843** ^**b**^	**0.2052 ± 0.1392** ^**b**^	**0.002**
Hospitals***		0.0045 ± 0.0041	0.0052 ± 0.0040	0.0035 ± 0.001	0.427	0.2748 ± 0.1525	0.2274 ± 0.0849	0.1902 ± 0.0466	0.062
Workplace‐worker		0.0031 ± 0.0028	0.0044 ± 0.0039	0.0027 ± 0.0021	0.201	0.247 ± 0.1267	0.2250 ± 0.0972	0.2282 ± 0.0968	0.902
Nursing home		0.0039 ± 0.0042	0.0039 ± 0.0041	0.0029 ± 0.0030	0.264	0.2874 ± 0.2198	0.3006 ± 1583	0.2279 ± 0.1064	0.142
Prison		0.0042 ± 0.0037	00038 ± 0.0030	0.0037 ± 0.0020	0.905	0.3080 ± 0.02150	0.2292 ± 0.133	0.1950 ± 0.0656	0.124
Preschool‐age	III‐course	0.0028 ± 0.0037	0.0035 ± 0.0044	0.0029 ± 0.0043	0.582	0.1285 ± 0.0841	0.2272 ± 0.1568	0.1966 ± 0.1185	0.072
Hospitals***		0.0030 ± 0.0031	0.0018 ± 0.0028	0.0024 ± 0.0030	0.051	0.1632 ± 0.0750	0.1375 ± 0.09303	0.2244 ± 0.1982	0.253
Workplace‐worker		0.0034 ± 0.0040	0.0042 ± 0.0053	0.0025 ± 0.0041	0.504	0.1888 ± 0.1532	0.2163 ± 0.1701	0.2066 ± 0.1582	0.718
Nursing home		0.0039 ± 0.0062	0.0021 ± 0.0028	0.0027 ± 0.0028	0.384	0.1207 ± 0.0906	01805 ± 01172	0.1647 ± 0.1020	0.137
Prison		0.0029 ± 0.0032	0.0027 ± 0.0050	0.2027 ± 0.0036	0.710	0.1402 ± 0.0466	0.2255 ± 0.1577	0.2022 ± 0.1370	0.091
Hospitals***	IV‐course	0.0025 ± 0.0029	0.0022 ± 0.0022	0.0032 ± 0.0035	0.274	**0.1942 ± 0.1118** ^**a**^	**0.2042 ± 0.1290** ^**a**^	**0.2904 ± 0.1758** ^**b**^	**0.039**

*Note:* Parameters with different exponent letters (a, b) are significantly different from each other. *p* < 0.05.

Abbreviations: DP, Digital Program Menus; IAM, internet‐accessible menus; NMPG, National Menu Planning Guidelines; SD, standard deviation.

* Kruskal‐Wallis Test.

*** Standard medical nutrition treatment, regime 3.

As shown in Table 3, the NRF 9.3 scores of II‐type meals of the DPM and NMPG menus, which target preschools (*p* = 0.030), nursing homes (*p* = 0.009) and prisons (*p* = 0.013), were higher than the NRF 9.3 score of menus with internet access for the same target groups. In addition, the NRF 9.3 score of the III‐type meals of the prison menus planned with the digital program was also found to be higher than that of the other prison menus (*p* < 0.001). When the menus were evaluated in terms of the SAIN‐LIM class score (as the score decreased, the nutritional value increased), the SAIN‐LIM scores of the I‐type and III‐type meals of the DPM targeting preschool‐age and the NMPG menus were similar (*p* > 0.05), but these scores were lower for I‐type meals than for menus with internet access (*p* < 0.001) and higher for III‐type meals (*p* = 0.027). The SAIN‐LIM class scores of the II‐type meals of the hospital menus planned with the digital program were similar to those of the NMPG menus and lower than those of the IAM (*p* = 0.032). The SAIN‐LIM class scores of the III‐type meals of the prison menus planned with the digital program were found to be higher than those of the other menu sources (*p* < 0.001).

**TABLE 3:** The mean NRF 9.3 and SAIN‐LIM class scores for menus.

Target group	Meal types	DPM	NMPG menus	IAM	*p* *	DPM	NMPG menus	IAM	*p* *
		Mean NRF 9.3 score ± SD				Mean SAIN‐LIM class score (Highest 1‐ Lowest 4) ± SD			
Preschool‐age	I‐course	2.5188 ± 1.2237	2.6900 ± 0.9703	2.6074 ± 0.7096	0.489	**2.90 ± 0.72** ^**a**^	**2.55 ± 0.94** ^**a**^	**1.5 ± 0.89** ^**b**^	**< 0.001**
Hospitals***		2.8592 ± 1.0714	2.9864 ± 1.7317	2.7098 ± 0.7208	0.985	2.68 ± 0.82	2.54 ± 0.92	2.43 ± 0.92	0.539
Workplace‐worker		2.6557 ± 1.0152	3.3464 ± 1.8990	3.7841 ± 4.0709	0.284	2.80 ± 0.62	2.40 ± 0.94	2.45 ± 1.00	0.322
Nursing home		3.0591 ± 1.2239	2.7410 ± 20.9448	2.8607 ± 1.7940	0.513	2.82 ± 0.67	2.57 ± 0.96	2.39 ± 1.20	0.494
Prison		2.5420 ± 1.0218	2.7637 ± 1.0095	2.7744 ± 0.8062	0.499	2.89 ± 0.74	2.32 ± 1.12	2.21 ± 1.20	0.073
Preschool‐age	II‐course	**2.1141 ± 2.0333** ^**a**^	**0.8655 ± 0.7267** ab	**0.8149 ± 0.9715** ^**b**^	**0.030**	2.20 ± 1.36	1.9 ± 1.02	1.85 ± 0.67	0.924
Hospitals***		1.3499 ± 1.2524	1.0258 ± 1.0403	1.3305 ± 1.4058	0.325	**2.07 ± 1.27** ab	**2.32 ± 1.28** ^**a**^	**1.46 ± 0.74** ^**b**^	**0.032**
Workplace‐worker		1.2578 ± 1.0943	1.3335 ± 1.0283	0.8941 ± 0.6879	0.304	2.00 ± 1.30	2.00 ± 1.34	1.90 ± 1.17	0.985
Nursing home		**1.5804 ± 1.6066** ^**a**^	**1.3736 ± 0.9427** ^**a**^	**0.7896 ± 0.5763** ^**b**^	**0.009**	1.71 ± 1.18	2.00 ± 1.25	1.96 ± 1.10	0.377
Prison		**1.5153 ± 1.4770** ^**a**^	**1.1036 ± 0.8716** ab	**0.7167 ± 0.5166** ^**b**^	**0.013**	2.21 ± 1.32	2.07 ± 1.21	1.61 ± 0.63	0.378
Preschool‐age	III‐course	1.2106 ± 1.3188	0.9068 ± 1.5572	0.4815 ± 1.4980	0.104	**1.50 ± 1.05** ^**a**^	**2.40 ± 1.35** ab	**2.55 ± 1.36** ^**b**^	**0.027**
Hospitals***		1.6393 ± 1.6622	1.3865 ± 1.7111	0.9002 ± 1.531	0.119	1.86 ± 1.30	1.71 ± 1.27	2.29 ± 1.44	0.252
Workplace‐worker		0.7868 ± 0.9153	1.0082 ± 1.6769	0.8467 ± 1.6720	0.594	2.00 ± 1.30	2.15 ± 1.35	2.25 ± 1.33	0.834
Nursing home		1.4849 ± 1.4574	1.2899 ± 1.5904	1.5382 ± 1.7306	0.849	1.46 ± 1.04	2.00 ± 1.31	1.82 ± 1.06	0.193
Prison		**1.8382 ± 1.3506** ^**a**^	**0.6540 ± 1.3473** ^**b**^	**1.0888 ± 1.7675** ^**b**^	**< 0.001**	**1.00 ± 0.00** ^**a**^	**2.57 ± 1.26** ^**b**^	**2.12 ± 1.29** ^**b**^	**< 0.001**
Hospitals***	IV‐course	1.3742 ± 0.9148	0.8960 ± 0.4947	1.0639 ± 0.5078	0.101	1.61 ± 1.03	1.75 ± 1.14	1.29 ± 0.85	0.104

*Note:* Parameters with different exponent letters (a, b) are significantly different from each other. *p* < 0.05.

Abbreviations: DP, Digital Programme Menus; IAM, internet accessible menus; NMPG, National Menu Planning Guidelines; SD, standard deviation.

* Kruskal‐Wallis Test.

^ab^ Mann‐Witney *U* Test.

*** Standard medical nutrition treatment, regime 3.

When the meal groups of the menus were evaluated in terms of cost (Table 4), the costs of the I‐type meals of the DPM targeting preschool age (*p* = 0.037) and prison (*p* = 0.024) were greater than those of the NMPG menus and IAM, whereas the costs of the workplace‐worker (*p* < 0.001) menus were lower. In addition, the costs of the III‐type meals of the prison menus planned with the digital program were similar to those of the NMPG menus but higher than those of the IAM (*p* = 0.002).

**TABLE 4:** The mean cost of one serving of menus ($).

Target group	Meal types	DPM	NMPG menus	IAM	*p* *
		Mean cost (per serving) ± SD			
Preschool‐age	I‐course	**1.52 ± 0.51** ^**a**^	**1.35 ± 0.74** ^**b**^	**1.12 ± 0.30** ab	**0.037**
Hospitals***		1.43 ± 0.53	1.6 ± 0.73	1.41 ± 0.51	0.359
Workplace‐worker		**1.50 ± 0.38** ^**a**^	**1.53 ± 0.64** ^**b**^	**1.51 ± 0.71** ^**b**^	**< 0.05**
Nursing home		1.34 ± 0.50	1.38 ± 0.70	1.17 ± 0.68	0.231
Prison		**1.56 ± 0.711** ^**a**^	**1.22 ± 0.45** ab	**1.12 ± 0.50** ^**b**^	**0.024**
Preschool‐age	II‐course	0.34 ± 0.27	0.23 ± 0.19	0.18 ± 0.07	0.134
Hospitals***		0.23 ± 016	0.26 ± 0.15	0.19 ± 0.11	0.548
Workplace‐worker		0.21 ± 0.21	0.23 ± 0.13	0.20 ± 0.20	0.139
Nursing home		0.26 ± 0.24	0.28 ± 0.18	0.19 ± 0.12	0.228
Prison		**0.26 ± 0.24** ^**a**^	**0.24 ± 0.17** ^**a**^	**0.13 ± 0.50** ^**b**^	**0.002**
Preschool‐age	III‐course	0.15 ± 0.12	0.20 ± 0.11	0.14 ± 0.10	0.207
Hospitals***		0.19 ± 0.10	0.15 ± 0.14	0.19 ± 0.22	0.186
Workplace‐worker		0.20 ± 0.13	0.16 ± 0.11	0.19 ± 0.15	0.742
Nursing home		0.14 ± 0.10	0.18 ± 0.15	0.17 ± 0.10	0.343
Prison		0.22 ± 0.36	0.19 ± 0.15	0.15 ± 0.07	0.679
Hospitals***	IV‐course	0.17 ± 0.10	0.16 ± 0.10	0.22 ± 0.14	0.478

*Note:* Parameters with different exponent letters (a, b) are significantly different from each other. *p* < 0.05.

Abbreviations: DP, Digital Programme Menus; IAM, internet accessible menus; NMPG, National Menu Planning Guidelines; SD, standard deviation.

* Kruskal‐Wallis Test.

^ab^ Mann‐Witney *U* Test.

*** Standard medical nutrition treatment, regime 3.

When each menu (1 month/lunch) was evaluated in terms of sustainability criteria based on the average of three types of meals (Table 5), the digital program and NMPG menus were similar in terms of their carbon footprint (kg/CO_2_ equivalent), water footprint (m^3^/ton), SAIN‐LIM class score and cost (*p* > 0.05). The NRF 9.3 score of the DPM was greater than those of both the NMPG menus and the IAM (*p* < 0.001). The carbon footprint (kg/CO_2_ equivalent) (*p* < 0.001), water footprint (m^3^/ton) (*p* < 0.001) and costs (*p* < 0.001) of the digital program and NMPG menus were greater than those of the IAM.

**TABLE 5:** Total sustainability criteria for menus.

Sustainability criteria	DPM	NMGP	IAM	*p* *
	(*n*: 5 menus)	(*n*: 5 menus)	(*n*: 5 menus)	
	(x̄ ±SS)	(x̄ ±SS)	(x̄ ±SS)	
Mean carbon footprint (kg/CO_2_ equivalent) (x̄ ±SS)	0.012 ± 0.110^a^	0.010 ± 0.004^ab^	0.009 ± 0.004^b^	**< 0.001**
‐ Mean total water footprint (m^3^/ton) (x̄ ±SS)	0.584 ± 0.215^a^	0.550 ± 0.211^ab^	0.521 ± 0.165^b^	**0.026**
Mean NRF 9.3 score (x̄ ±SS)	1.879 ± 0.697^a^	1.648 ± 0.626^b^	1.581 ± 0.831^b^	**< 0.001**
Mean SAIN‐LIM Class Score (Highest 1‐ Lowest 4) (x̄ ±SS)	2.095 ± 0.574	2.210 ± 0.654	2.005 ± 0.586	0.076
Mean cost ($ for one portion) (x̄ ±SS)	0.623 ± 0.244^a^	0.583 ± 0.222^a^	0.515 ± 0.196^b^	**< 0.001**

*Note:* Parameters with different exponent letters (a, b) are significantly different from each other. *p* < 0.05. *n* = 5 target groups × 3 sources: 15 menu/montly in total.

Abbreviations: DP, Digital Programme Menus; IAM, internet accessible menus; NMPG, National Menu Planning Guidelines; SD, standard deviation; x̄, Mean.

* Kruskal‐Wallis Test.

^ab^ Mann‐Witney *U* Test.

## 4 Discussion

This study aimed to develop a program for planning sustainable menus and to compare the environmental impacts, nutritional profiles, and costs of the menus planned from this program with those of the sample menus in the TNMPG and the menus of catering companies accessible on the internet. To the best of the authors' knowledge, this is the first study in which a program based on sustainability in FS has been developed and the relationships among menu quality, menu cost, carbon footprint, and water footprint with different menus, primarily the TNMPG, have been investigated. In addition, this study contributes to the comparison of various menus with national guides in studies on “sustainable nutrition” and “digitalization” in FS.

Menu planning constitutes the starting and control points of all activities in FS processes (Türkiye Ministry Health 2020). Therefore, it can be argued that the menu planning process is important for ensuring sustainability in all stages of FS. This study was planned and carried out under the assumption that a digital platform that enables the planning of sustainable menus in FS can also ensure sustainability in all processes.

Advances in technology have resulted in a very high increase in production in modern agriculture. These developments have also increased the negative environmental effects of food production and consumption (Baroni et al. 2007). To estimate the extent of these negative environmental effects that cause climate change, a tool is needed to monitor greenhouse gas emissions. The carbon footprint has become an easy tool for monitoring and measuring greenhouse gas emissions, as have programs carried out to reduce them (Caro 2019). In the presented study, the menu planning program, which was developed by considering the requirements of technology, also automatically calculates the sustainability criteria of the carbon footprint, water footprint, nutrient profiles, and costs.

The menus planned with this digital program are similar to other menus in terms of protein, carbohydrate, and energy values (*p* > 0.05), but they differ in the amount of food types they contain (Table 1).

In the present study, the carbon footprint (kg/CO_2_ equivalent) and water footprint (m^3^/ton) of I‐type meals were greater than those of meal types for all menus (Table 2). The main meals (I‐type meals) generally contained foods with high animal protein values, such as meat, chicken, and fish. It is a known fact that animal‐based foods (such as meat and meat products, milk and dairy products) have higher greenhouse gas emission values per kilogram than do plant‐based foods (such as fruits, vegetables, legumes, cereals), and the amount of water required for their production is greater per energy unit (Clune et al. 2017; Gerbens‐Leenes et al. 2013). Although the average (x̄ ±SD) carbon footprint of DPM is greater than that of other menus, it was determined that there was no significant difference between the carbon footprints of the food types in the menus obtained from three different sources for each target group (*p* > 0.05). The water footprint of all digitally planned menus, except for the II‐type meals of the DPM, whose target group is preschool‐age, is similar to that of the NMPG menus (*p* > 0.05, Table 2).

Similarly, in a study conducted by Volanti et al., the carbon footprint values of I‐type meals containing meat products and milk‐dairy products were high (Volanti et al. 2022). In a similar study conducted by Rosi et al., a web‐based application that supports sustainability was designed, and the nutritional values and environmental effects of the meals chosen by the workers were evaluated. As a result, this web design increased the choice of fish and plant‐based foods by 2% and the choice of whole grains by 17% (Rosi et al. 2022).

The current literature generally emphasizes that when present diets and eating habits are enriched with plant‐based foods, ecologically negative effects are minimized. However, considering current eating habits, without completely removing meat and dairy products from recipes and diets, consumption can be reduced by reducing them to reasonable levels without compromising the need for healthy nutrition, especially the negative environmental effects of main/meat meals (Chai et al. 2019).

Nutrient profiles are tools developed to evaluate food, menus and diet quality; classify them objectively according to their nutrient content; and assist consumers in food choices (Drewnowski 2010). The World Health Organization (WHO) has defined nutrient profiling as the science of classifying foods and beverages according to their nutrient composition (WHO 2011). In this study, nutrient profiles, which can be considered one of the sustainability criteria of menus, were calculated via the NRF 9.3 and SAIN‐LIM tools. In the presented study, the fact that the NRF 9.3 scores of III‐type meals, including salad, dessert, fruit, etc., in the prison menus planned with the digital program were higher than the scores of both the NMPG and menus accessible via the internet and lower than the SAIN‐LIM class scores is important in terms of showing the superiority of the DPM (*p* < 0.001, Table 3). “Added sugar”, “saturated fat” and “sodium” are among the nutrients that are requested to be restricted in the NRF 9.3 and SAIN‐LIM score algorithms. These results may be related to menus planned with digital programs, including “healthier milk and other desserts instead of ‘syrupy pastries with lots of added sugar’ and the fact that digitally programmed menus contain more ‘saturated fat’” in main dishes, especially red meat, milk, etc., than IAMs do and therefore have a higher cholesterol load.

In a study conducted by Delicado‐Soria et al., the NRF 9.3 and MDS values of traditional meals were directly proportional to each other out of the meals characterized into four groups: traditional meals, transitional meals, European breakfasts and Western meals (Delicado‐Soria et al. 2021). In fact, in a similar study conducted by Aytekin‐Sahin et al., five different hospital menus were not sustainable menus compared with the Mediterranean diet. They stated that this could be achieved by reducing the amount of red meat, which has a high carbon footprint and water footprint; replacing red meat with other protein sources, such as chicken, fish, and legumes; and increasing the number of vegetables and fruits (Aytekin‐Sahin et al. 2024).

According to the Food and Agriculture Organization (FAO), sustainable nutrition is defined as a diet that is nutritionally adequate, safe, healthy, economically affordable, and culturally acceptable and that minimizes environmental degradation on the planet (FAO 2023). In this context, sustainability also means cost, affordability of food, accessibility of meals, and reduction of waste/leftovers. In the present study, DPM was similar to NMPG menus in terms of cost, except for I‐type meals in preschool and workplace‐worker menus planned with the digital program (*p* > 0.05, Table 4). Type I meals include meat dishes, meatballs, meat‐vegetable dishes, meat‐legume dishes, and stuffed vegetables. The second group of dishes includes soup, rice, pasta, pastries, and olive oil dishes. Therefore, reducing the amount of high‐cost red meat in menus and increasing other quality protein sources, such as chickens, fish, and legumes, will reduce both negative environmental factors and costs and provide quality nutrition. A similar study conducted by Yacoub Bach et al. in 2023 reported that the diet based on WHO guidelines had the second largest negative environmental impact after the ketogenic diet and had the highest value of all diets in terms of cost (Yacoub Bach et al. 2023).

In conclusion, each menu (one‐month lunch/5 target group menus) was evaluated in terms of sustainability criteria based on the average of three types of meals in the present study (Table 1). The digital program and NMPG menus were statistically similar in terms of the carbon footprint (kg/CO_2_ equivalent), water footprint (m^3^/ton), SAIN‐LIM class score, and cost (*p* > 0.05). DPMs were found to have a higher NRF 9.3 score than other menus did (*p* < 0.001). The carbon footprint (kg/CO_2_ equivalent), water footprint (m^3^/ton) and costs of the digital program and NMPG menus were greater than those of the IAM (*p* < 0.001, Table 5). Although IAM has lower negative environmental impacts and costs, it has been shown that they are more negative in terms of nutrient profiles.

## 5 Conclusion

In conclusion, both DPM and NMPG menus need to maintain their nutrient profiles on the one hand, and on the other hand, the frequency of red meat in standard recipes in main dishes should be reviewed and revised in terms of protein‐based foods such as fish, chicken, and legumes. Moreover, when the DPM was compared with the NMPG menus, the DPM and NMPG menus were similar in terms of sustainability qualities. Therefore, in our study, it was shown that it is possible to plan sustainable menus in accordance with nutritional principles for FS and to evaluate the sustainability of these planned menus by calculating the ecological impact factors, nutritional pattern profiles, and costs in a short time. However, the recipes in the digital menu planning program used in the study consisted of traditional dishes found in Turkish cuisine. To increase the usability of the program in future studies, new recipes should be added by considering world cuisine, and the program should be further developed to maintain its up‐to‐date.

## Author Contributions

**Nida Nur Adiyan:** conceptualization (equal), data curation (equal), formal analysis (equal), investigation (equal), methodology (equal), resources (equal), software (equal), validation (equal), visualization (equal), writing – original draft (equal), writing – review and editing (equal). **Yasemin Beyhan:** conceptualization (equal), formal analysis (equal), methodology (equal), supervision (equal), validation (equal), writing – review and editing (equal). **Taygun Dayi:** formal analysis (equal), visualization (equal), writing – review and editing (equal).

## Ethics Statement

The study was conducted in accordance with the Declaration of Helsinki and approved by the Non‐interventional Scientific Research Ethics Committee of Hasan Kalyoncu University (Approval No: 2022/139 and 12 December 2022).

## Conflicts of Interest

The authors declare no conflicts of interest.

## Supporting information


**Data S1:** Supporting Information.

**Data S2:** Supporting Information.

**Data S3:** Supporting Information.

**Data S4:** Supporting Information.

**Data S5:** Supporting Information.

**Data S6:** Supporting Information.

## Data Availability

The raw data supporting the conclusions of this article will be made available by the authors upon request, without undue reservation.
