# Supplementary figures and images for: Evaluation of the Carbon Footprint, Water Footprint, Nutrient Profiles and Cost of Sustainable Menus Planned With Digital Modeling
Source: Food Sci Nutr. 2025 Sep 18;13(9):e70977. doi: 10.1002/fsn3.70977 (PMC12445120; doi:10.1002/fsn3.70977)

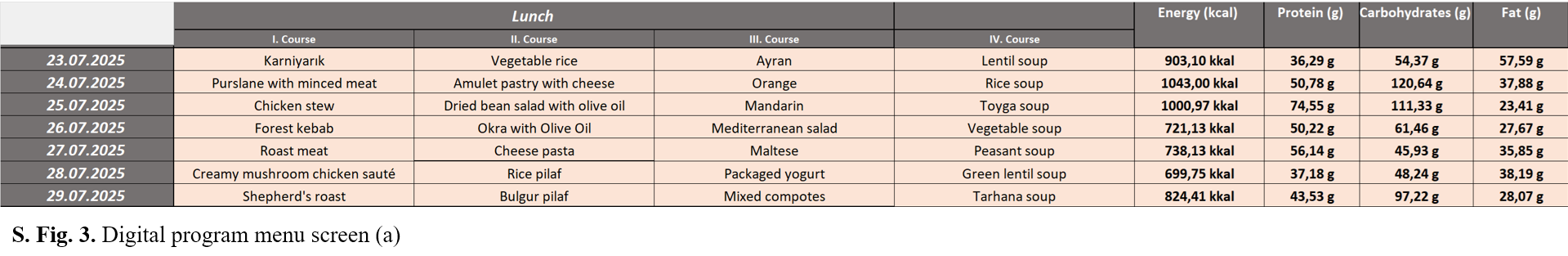

Supplement: Supplementary file 1 — Data S1: Supporting Information. [file FSN3-13-e70977-s001.png]

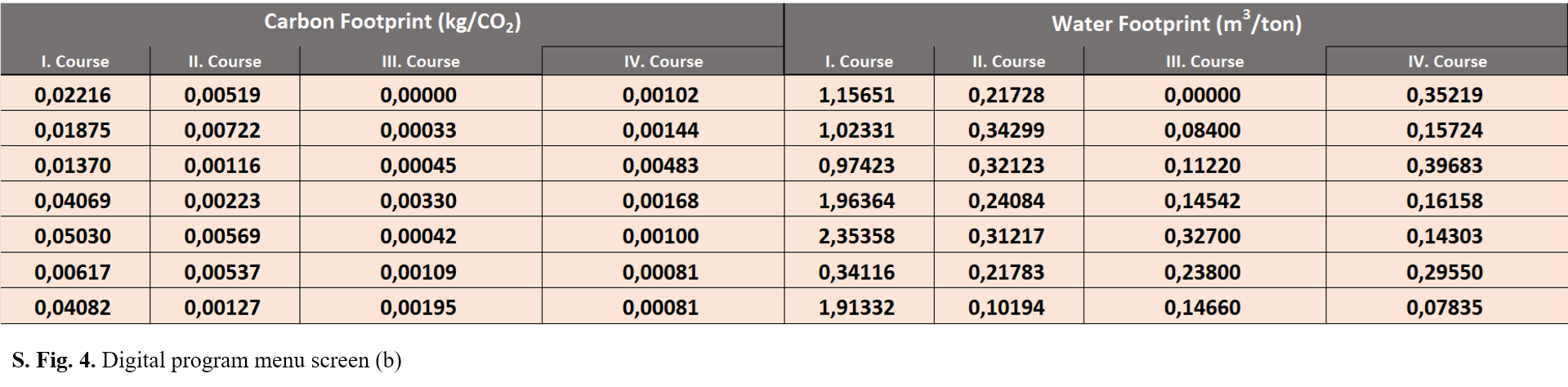

Supplement: Supplementary file 2 — Data S2: Supporting Information. [file FSN3-13-e70977-s004.png]

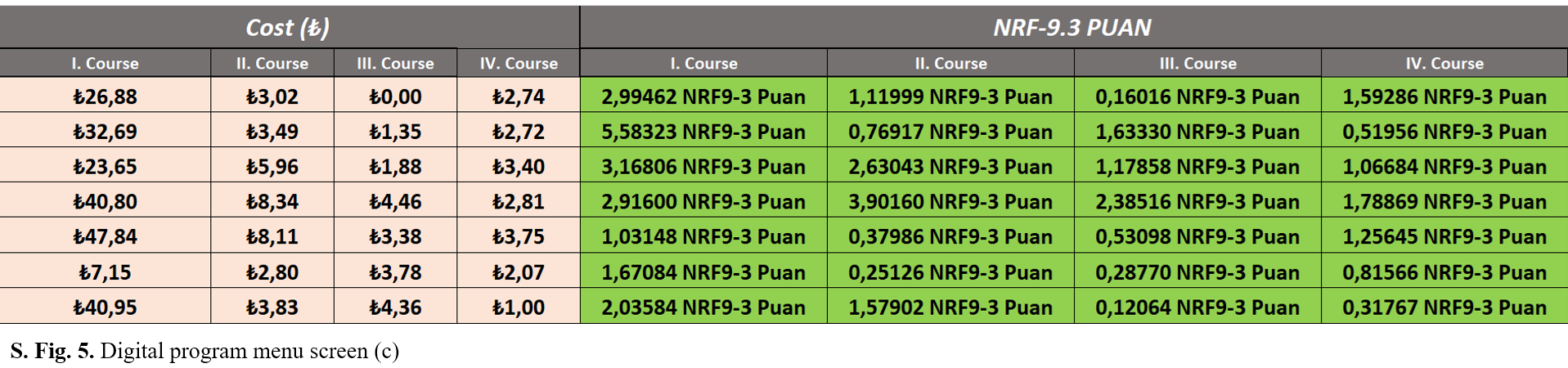

Supplement: Supplementary file 3 — Data S3: Supporting Information. [file FSN3-13-e70977-s003.png]

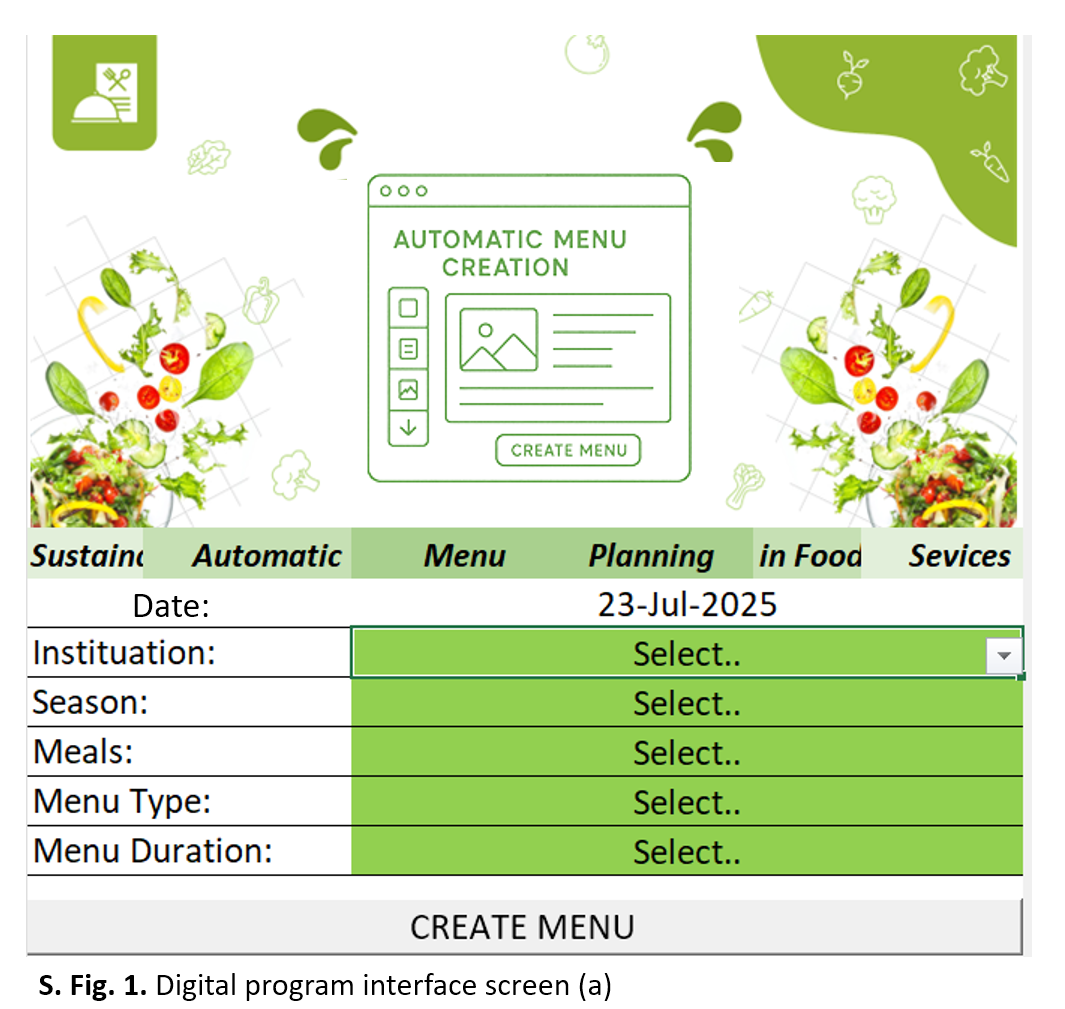

Supplement: Supplementary file 4 — Data S4: Supporting Information. [file FSN3-13-e70977-s006.png]

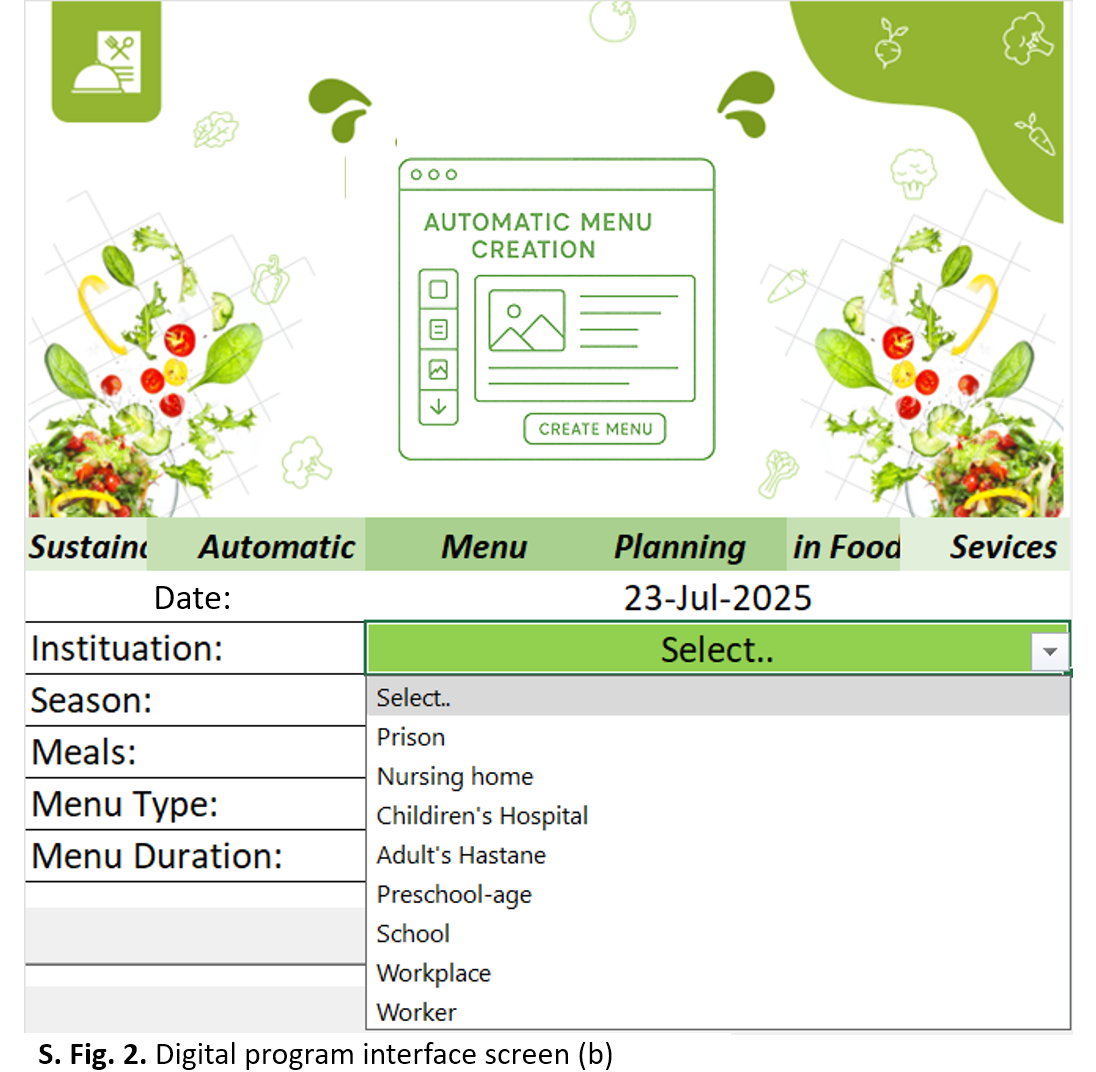

Supplement: Supplementary file 5 — Data S5: Supporting Information. [file FSN3-13-e70977-s005.png]

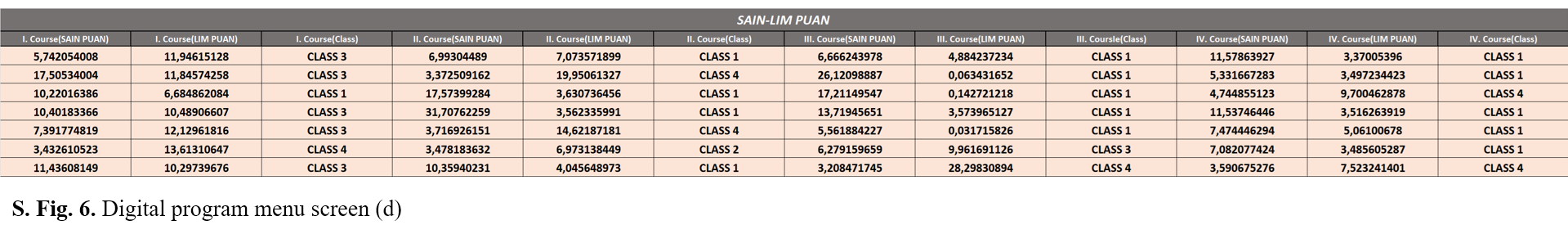

Supplement: Supplementary file 6 — Data S6: Supporting Information. [file FSN3-13-e70977-s002.png]
